# Evaluation of the Clinical Impact of Immune Checkpoint Inhibitors (Atezolizumab and Pembrolizumab) in the Treatment of Triple-Negative Breast Cancer: A Meta-Analysis and Review of Literature

**DOI:** 10.7759/cureus.98330

**Published:** 2025-12-02

**Authors:** Nafees Khan, Sidra Jabeen

**Affiliations:** 1 Internal Medicine, University Hospitals Sussex, Brighton, GBR; 2 Medicine and Surgery, Liaquat National Hospital and Medical College, Karachi, PAK

**Keywords:** atezolizumab, clinical outcomes, immune checkpoint inhibitors (icis), meta-analysis, pembrolizumab, triple-negative breast cancer (tnbc)

## Abstract

Triple-negative breast cancer (TNBC) refers to a distinct subclass of breast cancer with the absence of estrogen receptor (ER), progesterone receptor (PR), and human epidermal growth factor receptor 2 (HER2) expression, making it a clinically challenging subtype with fewer therapeutic pathways. Not only are these tumors unresponsive to receptor-targeted treatments, but also they typically manifest increased metastatic potential and invasiveness, high relapse rate, and poor outcomes. As such, there is a compelling clinical need for novel TNBC treatment regimens. A range of different therapeutic approaches are being investigated, including the use of immunotherapeutic agents. In this meta-analysis, we set out to evaluate the potential for using immune checkpoint inhibitors (ICIs), targeting either programmed cell death protein 1 (PD-1) or programmed cell death ligand 1 (PD-L1), in TNBC. A keyword search was performed using three databases, Cochrane Central Register of Controlled Trials (CENTRAL), Scopus, and PubMed, to identify potential studies to be included in a meta-analysis. Studies comparing the use of immune checkpoint inhibitors in combination with standard-of-care chemotherapy for the treatment of patients with TNBC were identified. The data from all the eligible studies were extracted, and statistical analysis was performed using Review Manager (RevMan) software (The Cochrane Collaboration, London, United Kingdom). Odds ratio (OR) and 95% confidence interval (CI) were calculated.

After filtering, six studies were included, containing a total of 4,824 patients. Four trials aimed to investigate the objective response rate (ORR), and three trials looked at the duration of response (DOR), all-cause adverse effects (AEs), and treatment-related adverse effects. The ORR was significantly higher in the PD-L1 inhibitor group (OR, 2.68; 95% CI, 2.09-3.44; p < 0.00001). Similarly, treatment with anti-PD-L1 therapy significantly increased the mean DOR (mean difference {MD}, 2.96; 95% CI, 2.74-3.18; p < 0.00001). All-cause adverse events and treatment-related adverse events were both higher in the anti-PD-L1 groups (OR: 1.93, 95% CI: 0.85-3.87, and p = 0.12 and OR: 1.66, 95% CI: 1.00-2.75, and p = 0.05, respectively). However, only treatment-related adverse events were significantly higher. This meta-analysis confirmed that regimens containing PD-1- or PD-L1-targeting agents have clinical utility and acceptable side-effect profiles when used in the treatment of triple-negative breast cancer. Future research should explore combination therapies to enhance efficacy and combat resistance.

## Introduction and background

Breast cancer has consistently remained the most prevalent malignancy in women, affecting 2.3 million women worldwide in 2020, with 685,000 deaths recorded in the same year [1], a figure that has steadily increased since 2012 [2]. Significant improvement in overall survival (OS) has greatly stalled since 2015, so there is an urgent need for alternative treatments [3]. Breast tumor cells that lack the expression of human epidermal growth factor receptor 2 (HER2), the progesterone receptor (PR), and the estrogen receptor (ER) define a subgroup of breast cancer as triple-negative breast cancer (TNBC) [4]. Improvements in response rates have been especially challenging to accomplish in the TNBC subgroup because these individuals have cancers with a strong metastatic potential, which leads to early recurrence rates and a poorer prognosis [5,6]. TNBC is also more common in younger age groups [7]. Premenopausal women below the age of 40 comprise 15%-20% of all patients with breast cancer [4]. Their survival time is shorter, with a five-year mortality rate of approximately 40% [4,8]. This contrasts with a 90% five-year survival for all breast cancer cases [9,10]. The difference in these expected outcomes highlights the need to develop more effective treatments for the population with TNBC.

TNBC has a high metastatic tendency due to its invasive nature, with around 46% of patients with TNBC developing a distant metastasis within three years of diagnosis. This contributes to an elevated post-surgery recurrence rate of 25% of distant metastasis, more often in the brain and visceral organs, and a median survival time of just 13.3 months [4]. Relapse rates in patients without TNBC vary between 35 and 67 months, as opposed to the shorter period, i.e., 19-40 months in patients with TNBC. Furthermore, patients with TNBC experience a high mortality rate of around 75% within three months following disease recurrence [4,11,12].

**Figure 1:**
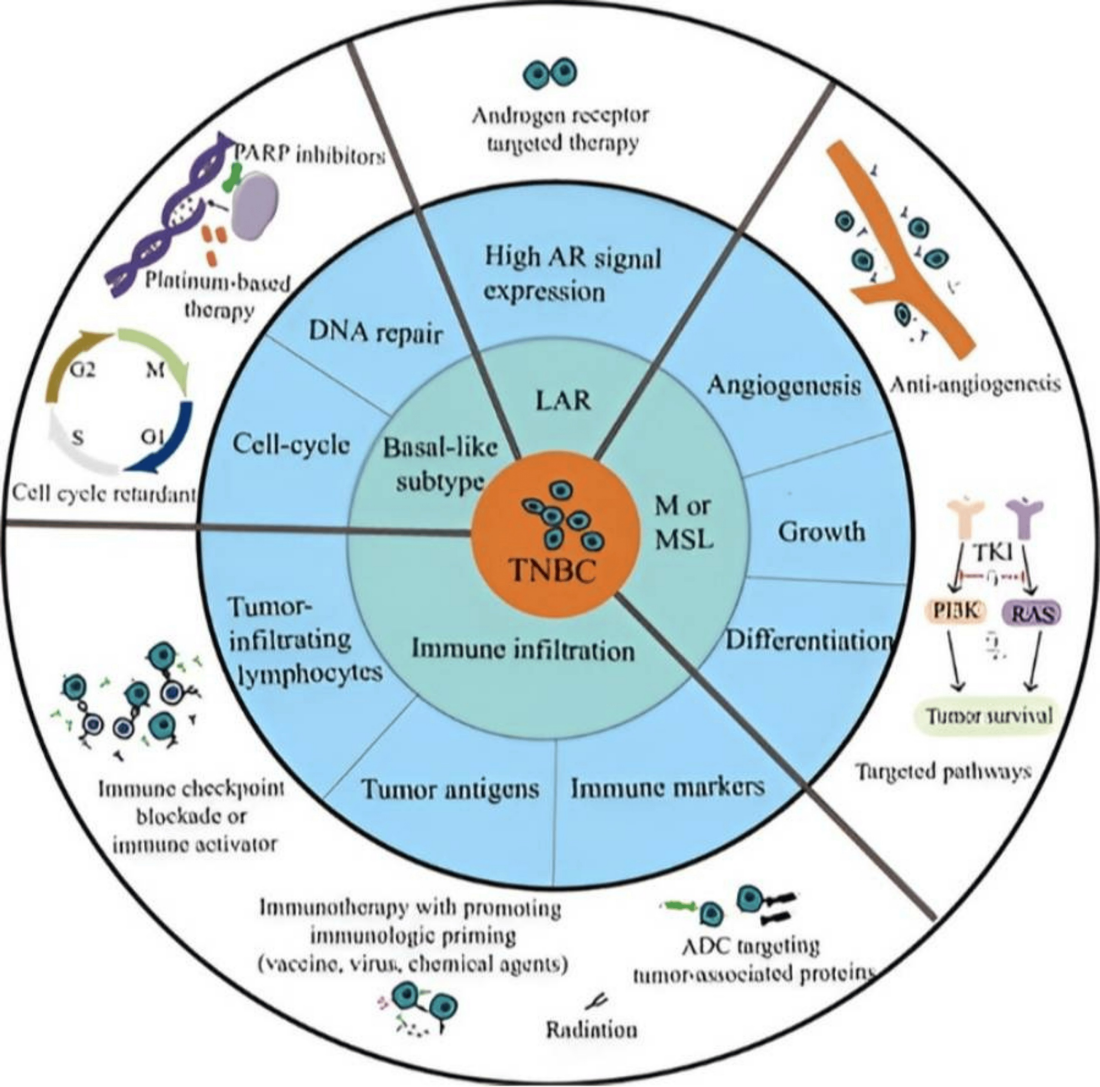
Classification and therapeutic options for TNBC Image sourced from “Recent advances in therapeutic strategies for triple-negative breast cancer,” by Li Y, Zhang H, Merkher Y, Chen L, Liu N, Leonov S, Chen Y, 2022, J Hematol Oncol, 15(1), 121 [21] under Creative Commons Attribution License CC BY 4.0 (https://creativecommons.org/licenses/by/4.0/). No changes were made ADC, antibody-drug conjugates; AR, androgen receptor; LAR, luminal androgen receptor; M, mesenchymal; MSL, mesenchymal-stem-like; PARP, poly-adenosine diphosphate ribose polymerase; PI3K, phosphoinositide-3 kinase; TKI, tyrosine kinase inhibitor; TNBC, triple-negative breast cancer

In response to these poor clinical outcomes, several novel therapeutic strategies have been developed for the treatment of patients with TNBC (Figure 1). These include immune checkpoint inhibitors (ICIs), antibody-drug conjugates (ADCs), and poly-adenosine diphosphate ribose polymerase (PARP) inhibitors [13-15]. In fact, gene mutation studies revealed that *BRCA1* or *BRCA2* gene mutations and/or deficits are present in around 15%-20% of patients with TNBC, which is significant since these defects play a critical role in compromising DNA integrity and encouraging carcinogenesis [16]. Importantly, the outcomes of practice-altering clinical studies using the PARP inhibitors, olaparib and talazoparib, confirmed their efficacy in patient groups with TNBC [17]. In terms of immunotherapy, while the introduction of ICIs has completely changed how many solid cancers, including metastatic TNBC (mTNBC), are treated, their role in the neoadjuvant therapy setting is still not entirely understood [18,19]. From a biological perspective, TNBC represents an immunogenic subtype of breast cancer since several preclinical studies have found that this disease has high levels of immune cell infiltrates and a high load of tumor mutations [20]. This should make them highly suited to immunotherapeutic interventions.

Standard of care in TNBC

Treatment approaches for stage I-III TNBC vary based on tumor size and lymph node involvement. Early-stage tumors may undergo breast-conserving surgery or mastectomy, often followed by adjuvant chemotherapy to reduce recurrence risk. For patients with *BRCA* mutations and specific tumor characteristics, such as larger size or lymph node involvement, olaparib may be added post-chemotherapy to improve outcomes. Neoadjuvant chemotherapy, sometimes with pembrolizumab, is used to shrink tumors before surgery, followed by additional treatments if residual cancer remains. In stage IV TNBC, initial therapy typically involves chemotherapy, with options such as anthracyclines, taxanes, or combinations thereof. Immunotherapy, particularly pembrolizumab for programmed cell death ligand 1 (PD-L1)-positive cancers, may be considered early in treatment. For refractory cases, PARP inhibitors or antibody-drug conjugates such as sacituzumab govitecan provide alternative options. Clinical trial participation is encouraged across all stages to explore novel therapies due to TNBC’s challenging prognosis and limited treatment options compared to other breast cancer types [22,23].

Immunotherapy for TNBC

TNBC is a good candidate for immunotherapeutic treatments, primarily because of tumor immune infiltration, neoantigens caused by higher genomic instability, and mutational burden, as well as high expression levels of immune checkpoint markers such as programmed cell death ligand 1 (PD-L1) and programmed cell death protein 1 (PD-1) [24]. These are highly correlated with tumor response, relapse, and overall outcomes [24]. Immunotherapy has shown success in a variety of neoplasms; consequently, immunotherapeutic approaches against TNBC offer great potential [25]. The first monoclonal antibody (mAb) licensed for TNBC, atezolizumab, was given FDA approval in 2019 for patients with metastatic TNBC that is unresectable, locally progressive, and positive for PD-L1. In 2020, the FDA additionally authorized the use of the anti-PD-1 antibody, pembrolizumab, when used in conjunction with chemotherapy for patients with similar indications with a combined positive score (CPS) of ≥10 [26].

In some solid tumors, high expression levels of the PD-L1 and PD-1 immune checkpoint molecules are known therapeutic targets [27]. According to reports, PD-1/PD-L1 are often expressed in breast tumors, particularly TNBC [28]. As PD-L1 from cancer cells may bind PD-1 on T cells, enabling cancer cells to avoid T cell-mediated immune recognition, PD-L1, PD-L2, and PD-1 are significant ICI molecules. Therefore, reactivating tumor-infiltrating lymphocytes (TILs) by blocking the PD-1/PD-L1 interaction with monoclonal antibodies can have beneficial therapeutic consequences in a variety of cancers, not just TNBC [13]. TNBC shows higher genomic instability and has the greatest mutational frequency of all breast cancer subtypes. Therefore, it may also produce neoantigens that may be detected by the immune system. These characteristics suggest that TNBC may be particularly amenable to treatment with immune checkpoint inhibitors.

Phase II and III trials have evaluated the efficacy of chemoimmunotherapy. In particular, two key studies, the KEYNOTE-522 [29] and the IMpassion031 [30], showed that the combination of immunotherapy and chemotherapy considerably enhanced the pathological complete response (pCR) as compared to chemotherapy alone [31]. However, it should be noted that a proportion of patients do not benefit from these strategies, as evidenced by the results of the GeparNuevo and NeoTRIP trials, which failed to meet their primary end points [30,32-34]. Nevertheless, there are still many unanswered questions regarding the role of neoadjuvant ICIs, including the absence of accurate predictors of response to TNBC immunotherapy. Here, we conducted a meta-analysis to evaluate the objective response rate (ORR), the duration of response (DOR), and adverse events in randomized controlled trials (RCTs) evaluating ICIs (atezolizumab and pembrolizumab) in combination with chemotherapy for patients with metastatic TNBC.

## Review

Methodology

Aim of the Study

Immune checkpoint inhibitors in combination with neoadjuvant therapy have been shown to be effective in multiple types of advanced cancers. In this study, we set out to evaluate the clinical efficacy and side-effect profiles associated with using atezolizumab and pembrolizumab as PD-1 or PD-L1 inhibitors in comparison to placebo and/or chemotherapy in the context of TNBC.

Search Strategy and Selection

This systematic review was carried out in accordance with the Preferred Reporting Items for Systematic Reviews and Meta-Analyses (PRISMA) statement and the Cochrane Handbook for Systematic Reviews of Interventions [35,36]. We retrieved relevant studies from 2007 to September 28, 2023, from the databases of Cochrane Central Register of Controlled Trials (CENTRAL), Scopus, Google Scholar, ClinicalTrials.gov, and PubMed, using the following search terms: “triple negative breast cancer OR metastatic triple negative breast cancer OR mTNBC OR TNBC” and “durvalumab OR atezolizumab OR nivolumab OR Bevacizumab OR pembrolizumab OR PD-L1 inhibitor OR PDL1 inhibitor OR immune checkpoint inhibitor.”

The literature search was conducted in accordance with the methodological standards of each database and search engine within a predefined data-lock period; therefore, studies published thereafter were not undertaken. The search yielded 134,193 relevant results.

Inclusion Criteria

**Figure 2:**
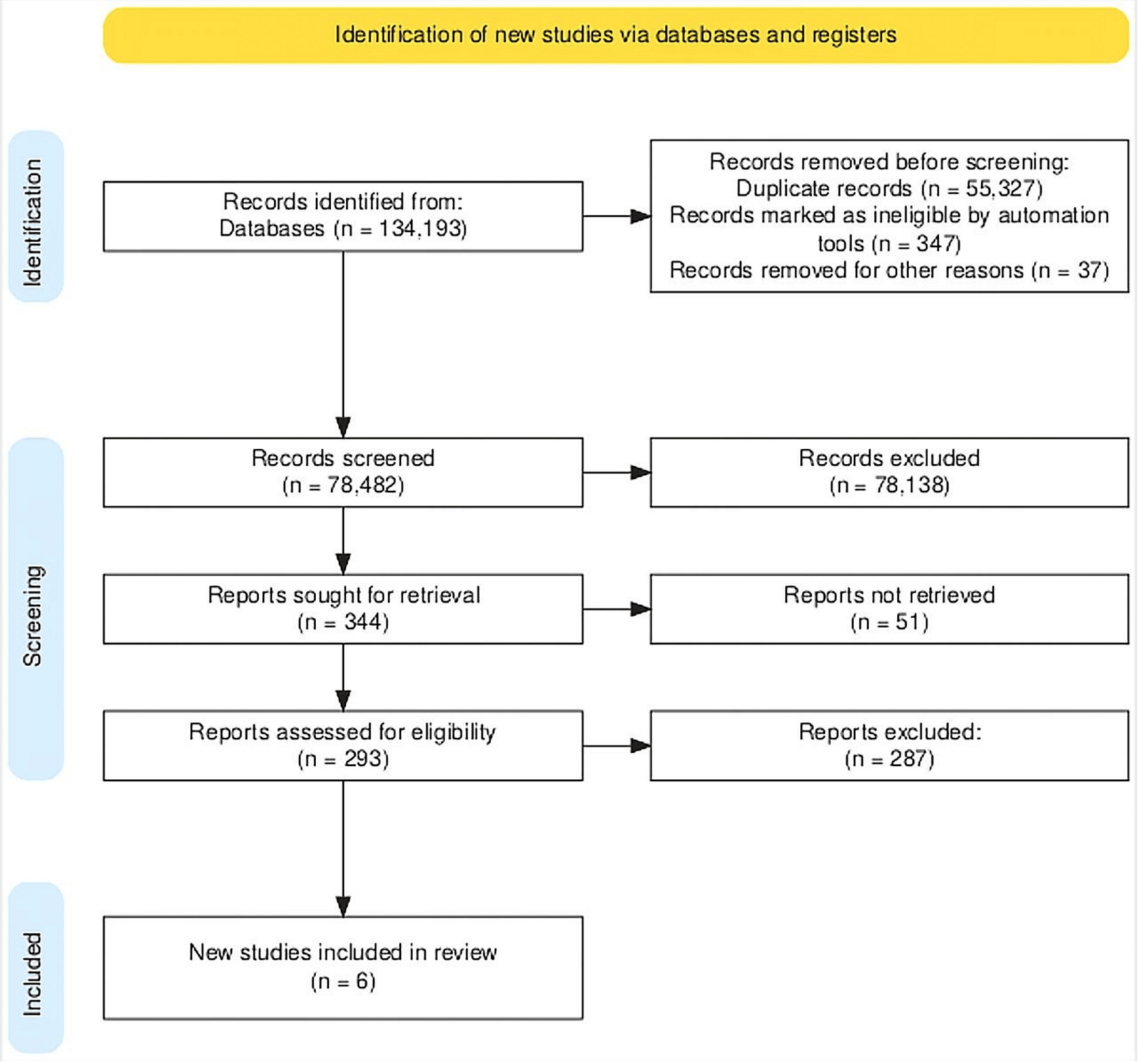
PRISMA flow diagram PRISMA flow diagram detailing our systematic search and selection process applied to include papers in our search PRISMA: Preferred Reporting Items for Systematic Reviews and Meta-Analyses

The search was restricted to human participants, and studies that were included in this meta-analysis were all available in English. Figure 2 illustrates the literature search approach in detail. Inclusion criteria are composed of the following: (i) study design: double-arm, randomized, phase III clinical trials; (ii) study population: patients over 18 years old with confirmed TNBC; (iii) language: English; (iv) intervention and comparison: PD-1 and/or PD-L1 inhibitor as either neoadjuvant or adjuvant therapy compared to placebo and/or chemotherapy; and (v) outcome parameter: ORR, DOR, adverse effects (AEs), and treatment-related adverse effects.

Exclusion Criteria

Exclusion criteria are composed of the following: (i) no extractable data, (ii) irretrievable full-text articles, (iii) single-arm trials, (iv) concomitant drug or comparator drug instead of placebo and/or conventional chemotherapy, and (v) non-phase III trials.

Data Extraction and Quality Assessment

**Figure 3:**
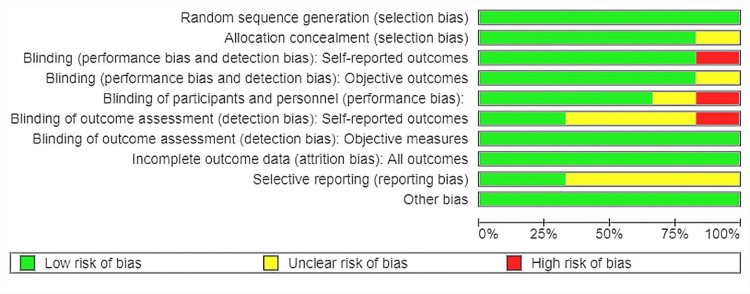
Risk of bias summary Risk of bias summary as generated by the Review Manager (RevMan) software (The Cochrane Collaboration, London, United Kingdom), showing different bias risk levels, with green being the lowest bias risk, yellow being the unclear bias risk, and red being the high bias risk

**Figure 4:**
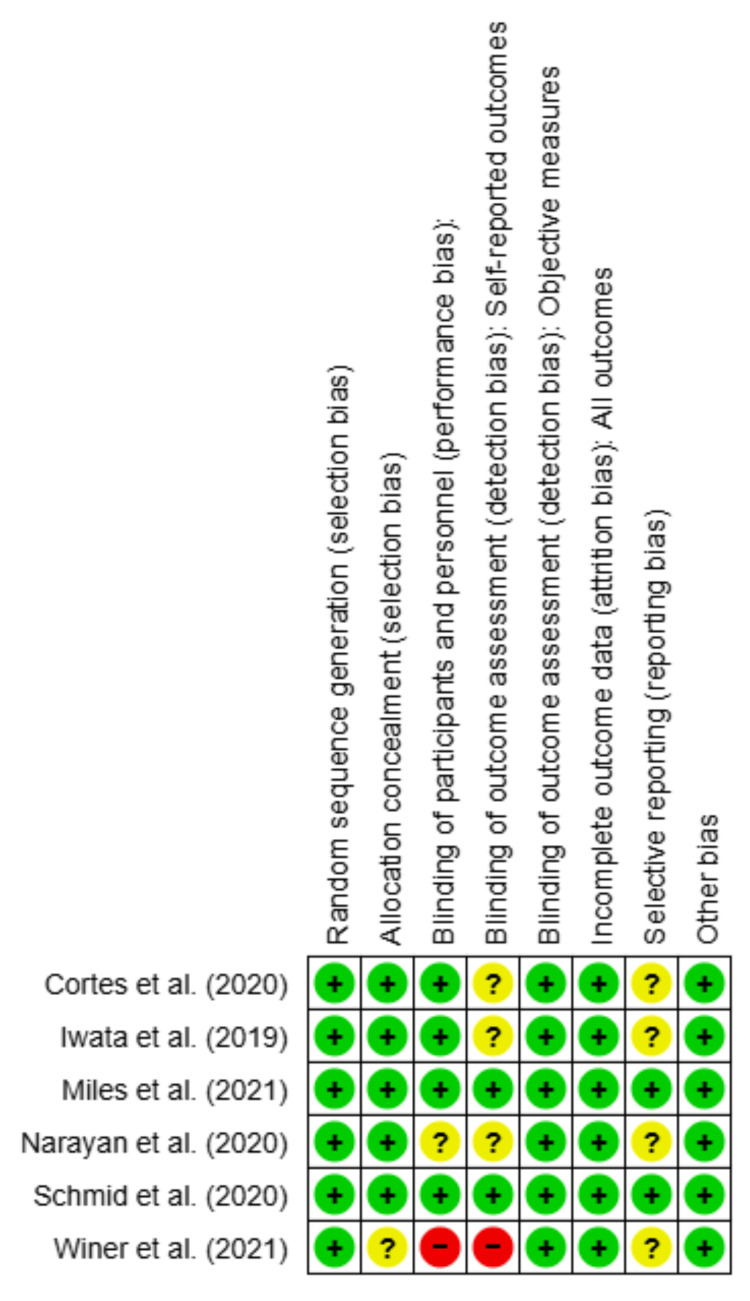
Risk of bias graph In the risk of bias graph, each row depicts a study, and the columns represent a type of bias [37-42]. The color indicates the risk of each bias type in each study, with green being the lowest bias risk, yellow being the unclear bias risk, and red being the high bias risk

Two independent reviewers (NK and SJ) initially evaluated the identified publications. The EndNote 20.0.1 program (Clarivate, London, United Kingdom) was used to filter the titles and abstracts and eliminate duplicates. We further validated the retrieved data through the process of title and abstract screening, full text screening, and data screening. The disagreements on the assessment of papers were resolved through mutual consensus. The study design, baseline parameters, and various outcomes were retrieved. The revised Cochrane risk of bias instrument (ROB-2, The Cochrane Collaboration, London, United Kingdom) was used to evaluate the quality of the included randomized controlled trials (RCTs). The quality evaluation of the included studies is described in detail in Figure 3 and Figure 4.

Study Definitions and End Points

Triple-negative breast cancer (TNBC) is defined as a breast cancer that does not express estrogen or progesterone receptors (ER or PR) and does not make the protein called human epidermal growth factor receptor 2 (HER2).

Programmed cell death protein 1 (PD-1) is an immune checkpoint protein expressed on cells of the immune system, including T cells. It normally acts as a type of “off switch” that helps keep the T cells from attacking other cells in the body. It does this when it binds to its ligand, PD-L1, a protein expressed on some normal (and cancer) cells.

PD-1 and PD-L1 inhibitors are a group of immune checkpoint inhibitors (ICIs) used in the treatment of multiple types of cancer; these include atezolizumab, durvalumab, pembrolizumab, and nivolumab. In our study, we limited the intervention to atezolizumab and pembrolizumab because of their superiority among the ICIs.

Objective response rate (ORR) is the percentage of people in a study or treatment group who have a partial response (PR) (>30% reversal of disease) or complete response (CR) (disease-free survival) to the treatment within a certain period of time.

The duration of response (DOR) is the time from randomization to disease progression or death in patients who achieve a complete or partial response. Partial regression is a decrease in the size of a tumor or in the extent of cancer in the body.

Complete regression is when all signs and symptoms of cancer have disappeared. All-cause adverse events are the adverse reactions seen commonly in response to the drugs given. Treatment-related adverse events are adverse effects seen particularly in response to the intervention drug given.

Statistical Analysis

We used the Review Manager (RevMan) computer program (version 5.4) to perform statistical analysis. The fixed effect (FE) model was used to keep the true effect size of the pooled studies. Additionally, changing the effect size to random effects (RE) produced similar effects, indicating that the populations across studies were not generalizable to a random population. The outcomes were reported as odds ratios (OR) with a 95% confidence interval (CI). For the assessment of study heterogeneity, the Higgins I-squared (I^2^) model was used with values of <25%, 25%-50%, 50%-75%, and >75% corresponding to no, low, moderate, and high degrees of heterogeneity, respectively [37-42]. A p value of <0.05 was considered statistically significant for all outcomes. The publication bias was depicted graphically using funnel plots where deemed necessary. However, due to a smaller number of trials in respective outcomes, Egger’s test was not conducted as it was not necessary. Mean differences (MD) were calculated from medians using mean-variance estimation.

Results

Literature Search

The preliminary literature search yielded 134,193 articles, of which 78,483 were screened for title and abstract using EndNote and Rayyan (Rayyan Systems, Inc., Cambridge, MA) as screening tools. Consequently, they were assessed for eligibility and full-text screening, which led to the inclusion of six studies with a total of 4,824 participants [37-42]. However, the total population varied for each outcome based on the reporting by the trials. The quality assessment of included studies is summarized in Figure 3 and Figure 4.

Study Characteristics

The patient characteristics of the included studies are shown in Table 1. A pooled total of 4,824 patients was included in our systematic review and meta-analysis. The trials were published between 2019 and 2021. Four trials aimed to investigate the ORR, and three trials looked at DOR, adverse effects, and treatment-related adverse effects, which were also the primary end points of our study.

**Table 1:** Baseline characteristics of the included studies Included studies are found in the column with characteristics in rows stated for intervention versus control [37-42] N, total population; n, subset population; ECOG, Eastern Cooperative Oncology Group; PD-L1, programmed cell death ligand 1; ITT, intention to treat

Characteristics of studies				Narayan et al. (2020) [37]		Schmid et al. (2020) [40]		Iwata et al. (2019) [39]	Miles et al. (2021) [38]		Winer et al. (2021) [42]	Cortes et al. (2020) [41]		
				N = 900		N = 902		N = 902	N = 651		N = 622	N = 847		
Sample size				ITT (n = 451)	PD-L1+ (n = 185)	ITT (n = 902)	PD-L1+ (n = 369)	ITT (n = 65)	ITT (n = 651)	PD-L1+ (n = 292)	ITT (n = 312)	PD-L1+ > 10 (n = 323)	PD-L1+ > 1 (n = 636)	ITT (n = 847)
Intervention				Atezolizumab + paclitaxel protein-bound (n = 451)	Atezolizumab + paclitaxel protein-bound (n = 185)	Atezolizumab + nab-paclitaxel (n = 450)	Atezolizumab + nab-paclitaxel group (n = 185)	Atezolizumab + nab-paclitaxel	Atezolizumab-paclitaxel (n = 431)	Atezolizumab-paclitaxel (n = 191)	Pembrolizumab (n = 312)	Pembrolizumab-chemotherapy (n = 566)	-	-
Control				Placebo + paclitaxel protein-bound (n = 451)	Placebo + paclitaxel protein-bound (n = 184)	placebo + nab-paclitaxel (n = 451)	Placebo + nab-paclitaxel group (n = 184)	Placebo + nab-paclitaxel (n = 31)	Placebo-paclitaxel (n = 220)	Placebo-paclitaxel (n = 101)	Chemotherapy (n = 310)	Placebo-chemotherapy (n = 281)	-	-
Age, mean (range)		Intervention		54.3 (42-66)	53.7 (40-66)	55 (46-64)	53 (44-63)	55.0 (31-82)	54 (22-85)	55 (23-83)	50 (43-59)	52 (44-62)	-	-
		Control		55.4 (43-67)	53.6 (41-65)	56 (47-65)	53 (44-63)	64.0 (37-77)	53 (25-81)	53 (25-78)	53 (44-61)	55 (43-63)	-	-
Sex, n (%)	Female	Intervention		448 (>99)	184 (>99)	448 (99)	184 (99)	34 (100)	430 (100)	191 (100)	312 (100)	-	-	-
		Control		450 (>99)	184 (100)	450 (>99)	184 (100)	31 (100)	220 (100)	101 (100)	308 (99)	-	-	-
	Male	Intervention		3	1	3 (1)	1 (1)	-	1 (<1)	0	0	-	-	-
		Control		1	0	1 (<1)	0	-	0	0	2 (1)	-	-	-
Baseline ECOG, n (%)	0	Intervention		256 (56.9)	107 (57.8)	256 (57)	107 (58)	28 (82.4)	262 (61)	118 (62)	169 (54)	134 (61)	253 (60)	332 (59)
		Control		270 (60)	112 (60.9)	270 (60)	112 (61)	27 (87.1)	191 (100)	59 (58)	158 (51)	62 (60)	134 (64)	173 (62)
	1	Intervention		193 (42.9)	77 (41.6)	193 (43)	77 (42)	6 (17.6)	169 (39)	73 (38)	141 (45)	86 (39)	171 (40)	232 (41)
		Control		179 (39.8)	72 (39.1)	179 (40)	72 (39)	4 (12.9)	90 (41)	42 (42)	151 (49)	41 (40)	77 (36)	108 (38)
	2	Intervention		1 (0.2)	1 (0.5)	1 (<1)	1 (1)	-	-	-	1 (<1)	0	0	1 (<1)
		Control		1 (0.2)	0 (0)	1 (<1)	0	-	-	-	0	0	0	0
Metastatic disease, n (%)		Intervention		-	-	-	-	32 (94.1)	-	-	66 (21)	144 (65)	274 (64)	383 (68)
		Control		-	-	-	-	22 (71.0)	-	-	64 (21)	144 (65)	135 (64)	185 (66)
Number of sites of metastatic disease, n (%)	0-3	Intervention		332 (73.8)	149 (80.5)	1 (1)	149 (81)	27 (79.4)	-	-	-	122 (55)	232 (55)	313 (55)
		Control		341 (75.9)	140 (76.5)	341 (76)	140 (77)	25 (80.6)	-	-	-	62 (60)	130 (62)	166 (59)
	>4	Intervention		118 (26.2)	36 (19.5)	118 (26)	36 (19)	7 (20.6)	105 (24)	35 (18)	-	97 (44)	190 (45)	250 (44)
		Control		108 (24.1)	43 (23.5)	108 (24)	43/183 (23)	6 (19.4)	48 (22)	105 (24)	-	41 (40)	81 (38)	115 (41)
Site of metastatic disease, n (%)	Liver	Intervention	Yes	126 (27.9)	42 (22.7)	126 (28)	44 (24)	11 (32.4)	-	-	-	62 (28)	131 (31)	171 (30)
			No	325 (72.1)	143 (77.3)	-	-	-	61 (28)	24 (24)	-	-	-	-
		Control	Yes	118 (26.2)	41 (22.3)	118 (26)	39 (21)	6 (19.4)	-	-	-	32 (31)	61 (29)	78 (28)
			No	333 (73.8)	143 (77.7)	-	-	-	118 (27)	37 (19)	-	-	-	-
	Bone	Intervention	Yes	145 (32.2)	54 (29.2)	145 (32)	54 (29)	7 (20.6)	-	-	-	52 (24)	112 (26)	169 (30)
			No	306 (67.8)	131 (70.8)	-	-	-	60 (27)	18 (18)	-	-	-	-
		Control	Yes	141 (31.3)	49 (26.6)	141 (31)	49 (27)	9 (29.0)	-	-	-	22 (21)	54 (26)	85 (30)
			No	310 (68.7)	135 (73.4)	-	-	-	140 (32)	44 (23)	-	-	-	-
	Brain	Intervention	Yes	30 (6.7)	15 (8.1)	30 (7)	15 (8)	1 (2.9)	-	-	20 (6)	5 (2)	14 (3)	17 (3)
			No	421 (93.3)	170 (91.9)	-	-	-	-	-	292 (94)	-	-	-
		Control	Yes	31 (6.9)	11 (6)	31 (7)	11 (6)	0	-	-	22 (7)	6 (6)	8 (4)	9 (3)
			No	420 (93.1)	173 (94)	-	-	-	-	-	288 (93)	-	-	-
	Lung	Intervention	Yes	173 (94)	86 (46.5)	226 (51)	86 (46)	16 (47.1)	-	-	-	120 (55)	236 (56)	324 (57)
			No	225 (49.9)	99 (53.5)	-	-	-	-	-	-	-	-	-
		Control	Yes	242 (53.7)	98 (53.3)	242 (54)	98 (53)	12 (38.7)	-	-	-	55 (53)	119 (56)	162 (58)
			No	209 (46.3)	86 (46.7)	-	-	-	-	-	-	-	-	-
	Lymph node	Intervention	Yes	33 (7.3)	18 (9.7)	33 (7)	18 (10)	3 (8.8)	-	-	-	169 (77)	318 (75)	417 (74)
			No	417 (92.7)	167 (90.3)	-	-	-	-	-	-	-	-	-
		Control	Yes	23 (5.1)	13 (7.1)	23 (5)	13 (7)	3 (8.8)	-	-	-	79 (77)	157 (74)	206 (73)
			No	426 (94.9)	170 (92.9)	-	-	-	-	-	-	-	-	-
Previous therapy, n (%)	Neo/adjuvant	Intervention		284 (63)	125 (67.6)	284 (63)	125 (68)	19 (55.9)	-	-	-	46 (21)	91 (21)	124 (22)
		Control		286 (63.4)	117 (63.6)	286 (63)	117 (64)	11 (35.5)	-	-	-	19 (18)	45 (21)	62 (22)
	Taxane	Intervention		-	-	231 (51)	96 (52)	15 (44.1)	208 (48)	97 (51)	-	107 (49)	213 (50)	290 (51)
		Control		-	-	230 (51)	94 (51)	11 (35.5)	107 (49)	54 (53)	-	50 (49)	115 (55)	156 (56)
	Anthracycline	Intervention		243 (53.9)	109 (58.9)	243 (54)	109 (59)	17 (50.0)	212 (49)	98 (51)	-	115 (52)	227 (53)	318 (56)
		Control		242 (53.7)	101 (54.9)	242 (54)	101 (55)	11 (35.5)	110 (50)	50 (50)	-	50 (49)	115 (55)	155 (55)

Table 2 and Table 3 show the treatment-related and immune-mediated adverse effects. The frequency of adverse events in patients treated with associated PD-1 or PD-L1 inhibitors was similar to that observed in the chemotherapy comparator arms. However, there were apparent differences in the immune-mediated adverse effects (Table 3), which may be related to the mechanism of action of these agents and/or because the PD-1/PD-L1-treated groups received an extra therapeutic agent.

**Table 2:** Adverse effects and treatment-related adverse effects Adverse effects and treatment-related adverse effects in the included studies. Included studies found in the column with different adverse effects found in rows AST, aspartate aminotransferase; ALP, alkaline phosphatase; LDH, lactate dehydrogenase; LFT, liver function test

Treatment-related adverse events	Winer et al. (2021) [42]				Iwata et al. (2019) [39]				Miles et al. (2021) [38]				Cortes et al. (2020) [41]			
	Pembrolizumab group (n = 309)		Chemotherapy group (n = 292)		Atezolizumab + nab-paclitaxel (n = 34)		Placebo + nab-paclitaxel (n = 30)		Atezolizumab + paclitaxel (n = 432)		Placebo + paclitaxel (n = 217)		Pembrolizumab-chemotherapy group (n = 562)		Placebo-chemotherapy group (n = 281)	
	Any grade	Grade ≥ 3	Any grade	Grade ≥ 3	Any grade	Grade ≥ 3	Any grade	Grade ≥ 3	Any grade	Grade ≥ 3	Any grade	Grade ≥ 3	Any grade	Grade ≥ 3	Any grade	Grade ≥ 3
Fatigue	37	3	41	3	8	-	6	-	-	-	-	-	16	16	83	7
Nausea	31	0	63	1	16	-	12	1	-	-	-	-	221	9	115	4
Vomiting	-	-	-	-	7	1	3	-	-	-	-	-	-	-	-	-
Anemia	-	-	-	-	-	-	-	-	-	-	-	-	541	383	267	188
Hypothyroidism	23	1	-	-	4	-	-	-	-	-	-	-	-	-	-	-
Pruritus	21	1	8	-	6	-	2	-	-	-	-	-	-	-	-	-
Diarrhea	18	2	46	8	6	-	5	-	-	-	-	-	-	-	-	-
Pyrexia	17	1	14	1	8	-	6	-	-	-	-	-	-	-	-	-
Asthenia	19	4	25	3	-	-	-	-	-	-	-	-	-	-	-	-
Decreased appetite	14	-	34	1	7	-	8	1	-	-	-	-	-	-	-	-
Headache	14	1	13	-	6	-	4	-	-	-	-	-	-	-	-	-
Increased AST	20	8	21	4	4	-	5	1	-	-	-	-	-	-	-	-
Constipation	12	-	29	1	8	-	10	-	-	-	-	-	-	-	-	-
Dyspnea	13	1	8	-	-	-	-	-	-	-	-	-	-	-	-	-
Increased ALP	13	3	20	1	5	-	8	1	-	-	-	-	115	33	46	13
Malaise	9	1	11	-	5	-	10	-	-	-	-	-	-	-	-	-
Myalgia	8	-	9	1	6	-	7	1	-	-	-	-	-	-	-	-
Maculopapular rash	6	1	8	1	10	-	6	-	141	4	66	2	-	-	-	-
Abdominal pain	5	1	8	1	-	-	-	-	-	-	-	-	-	-	-	-
Increased blood LDH	6	2	7	-	-	-	-	-	-	-	-	-	-	-	-	-
Peripheral sensory neuropathy	4	-	19	3	20	-	15	-	-	-	-	-	-	-	-	-
Leukopenia	3	-	7	5	-	-	-	-	-	-	-	-	-	-	-	-
Pneumonitis	5	2	-	-	-	-	-	-	16	3	3	-	-	-	-	-
Stomatitis	3	-	21	1	9	-	3	-	-	-	-	-	-	-	-	-
Decreased WBC count	4	-	35	16	10	4	3	2	-	-	-	-	-	-	-	-
Alopecia	2	-	39	-	34	13	30	12	-	-	-	-	186	5	94	3
Chest pain	2	-	1	1	-	-	-	-	-	-	-	-	-	-	-	-
Lethargy	3	1	5	-	-	-	-	-	-	-	-	-	-	-	-	-
Lymphopenia	4	2	3	-	-	-	-	-	-	-	-	-	-	-	-	-
Decreased neutrophil count	3	1	62	35	15	6	10	5	-	-	-	-	125	98	74	57
Pneumonia	2	-	3	2	-	-	-	-	-	-	-	-	-	-	-	-
Weight decrease	3	1	9	2	-	-	-	-	-	-	-	-	-	-	-	-
Abdominal distension	1	-	2	1	-	-	-	-	-	-	-	-	-	-	-	-
Neutropenia	1	-	86	52	4	3	-	-	-	-	-	-	231	167	107	84
Palmar-plantar erythrodysesthesia	2	-	36	7	-	-	-	-	-	-	-	-	-	-	-	-
Secondary adrenocortical insufficiency	2	1	-	-	-	-	-	-	-	-	-	-	-	-	-	-
Thrombocytopenia	2	1	7	1	-	-	-	-	-	-	-	-	-	-	-	-
Urticaria	2	1	1	-	-	-	-	-	-	-	-	-	-	-	-	-
Enteritis	-	-	2	1	-	-	-	-	-	-	-	-	-	-	-	-
Increased gamma-glutamyl transferase	1	1	5	1	-	-	-	-	-	-	-	-	-	-	-	-
Decreased lymphocyte count	-	-	7	4	-	-	-	-	-	-	-	-	-	-	-	-
Mucosal inflammation	-	-	22	4	-	-	-	-	-	-	-	-	-	-	-	-
Pharyngotonsillitis	-	-	2	1	2	-	3	-	-	-	-	-	-	-	-	-
Urinary tract infection	-	-	7	1	-	-	-	-	-	-	-	-	-	-	-	-
Asthma	1	1	-	-	-	-	-	-	-	-	-	-	-	-	-	-
Axillary pain	1	1	-	-	-	-	-	-	-	-	-	-	-	-	-	-
Brain edema	1	1	-	-	-	-	-	-	-	-	-	-	-	-	-	-
Hepatotoxicity	1	1	-	-	5	-	8	-	-	-	-	-	-	-	-	-
Hyponatremia	1	1	1	-	-	-	-	-	-	-	-	-	-	-	-	-
Interstitial lung disease	1	1	-	-	-	-	-	-	-	-	-	-	-	-	-	-
Liver disorder	1	1	-	-	2	-	-	-	-	-	-	-	-	-	-	-
Myositis	1	1	-	-	-	-	1	-	2	-	-	-	-	-	-	-
Pleural effusion	1	1	1	1	-	-	-	-	-	-	-	-	-	-	-	-
Supraventricular tachycardia	1	1	-	-	-	-	-	-	-	-	-	-	-	-	-	-
Type 1 diabetes	1	1	-	-	-	-	-	-	-	-	-	-	-	-	-	-
Acute kidney injury	-	-	1	1	-	-	-	-	-	-	-	-	-	-	-	-
Decreased blood magnesium	-	-	1	1	-	-	-	-	-	-	-	-	-	-	-	-
Colitis	-	-	1	1	-	-	-	-	3	1	2	2	-	-	-	-
Febrile neutropenia	-	-	10	10	-	-	-	-	-	-	-	-	-	-	-	-
Increased LFT	-	-	1	1	-	-	-	-	-	-	-	-	-	-	-	-
Mastitis	-	-	1	1	-	-	-	-	-	-	-	-	-	-	-	-
Myelodysplastic syndrome	-	-	1	1	-	-	-	-	-	-	-	-	-	-	-	-
Pancytopenia	-	-	2	2	-	-	-	-	-	-	-	-	-	-	-	-
Pulmonary embolism	-	-	1	1	-	-	-	-	-	-	-	-	-	-	-	-
Tonsillitis	-	-	1	1	-	-	-	-	-	-	-	-	-	-	-	-
Rhabdomyolysis	1	1	-	-	-	-	-	-	2	-	-	-	-	-	-	-
Systemic candida	-	-	1	1	-	-	-	-	-	-	-	-	-	-	-	-
Circulatory collapse	1	1	-	-	-	-	-	-	-	-	-	-	-	-	-	-
Hemothorax	-	-	1	1	-	-	-	-	-	-	-	-	-	-	-	-
Sepsis	-	-	1	1	-	-	-	-	-	-	-	-	-	-	-	-
Hepatitis (laboratory abnormalities)	-	-	-	-	5	-	8	-	-	-	-	-	-	-	-	-
Hepatitis (diagnosis)	-	-	-	-	2	-	-	-	7	2	2	-	-	-	-	-
Pancreatitis	-	-	-	-	-	-	-	-	9	7	1	1	-	-	-	-
Ocular inflammatory toxicity	-	-	-	-	-	-	-	-	4	-	1	-	-	-	-	-

**Table 3:** Immune-mediated adverse effects and treatment-related adverse effects Immune-mediated adverse effects and treatment-related adverse effects in the included studies. Included studies found in the column with different adverse effects found in rows

Treatment-related adverse events	Winer et al. (2021) [42]				Iwata et al. (2019) [39]				Miles et al. (2021) [38]				Cortes et al. [41]			
	Pembrolizumab group (n = 309)		Chemotherapy group (n = 292)		Atezolizumab + nab-paclitaxel (n = 34)		Placebo + nab-paclitaxel (n = 30)		Atezolizumab + paclitaxel (n = 432)		Placebo + paclitaxel (n = 217)		Pembrolizumab-chemotherapy group (n = 562)		Placebo-chemotherapy group (n = 281)	
	Any grade	Grade ≥ 3	Any grade	Grade ≥ 3	Any grade	Grade ≥ 3	Any grade	Grade ≥ 3	Any grade	Grade ≥ 3	Any grade	Grade ≥ 3	Any grade	Grade ≥ 3	Any grade	Grade ≥ 3
Hypothyroidism	24	1	4	-	6	-	1	-	60	-	12	-	87	2	9	-
Hyperthyroidism	11	-	-	-	2	-	-	-	25	-	-	-	27	1	3	-
Pneumonitis	6	3	-	-	1	-	-	-	-	-	-	-	14	6	-	-
Severe skin reaction	5	2	1	1	15	-	9	-	-	-	-	-	10	10	1	-
Adrenal insufficiency	3	1	-	-	-	-	-	-	3	-	-	-	-	-	-	-
Colitis	1	-	2	1	-	-	-	-	-	-	-	-	10	2	4	-
Myasthenia syndrome	1	-	-	-	-	-	-	-	1	-	-	-	-	-	-	-
Nephritis	1	-	-	-	-	-	-	-	2	1	1	-	-	-	-	-
Meningitis	-	-	-	-	-	-	-	-	2	1	1	-	-	-	-	-
Thyroiditis	1	-	1	-	-	-	-	-	-	-	-	-	-	-	-	-
Myositis	3	3	-	-	-	-	1	-	2	-	-	-	-	-	-	-
Type 1 diabetes	1	1	1	1	-	-	-	-	5	4	2	2	-	-	-	-
Infusion-relayed reactions	-	-	-	-	-	-	-	-	15	3	7	-	-	-	-	-

Interventions: Intervention group comprised either atezolizumab or pembrolizumab + nab-paclitaxel ± chemotherapy, as compared to the control group of control + nab-paclitaxel ± chemotherapy. Atezolizumab is an anti-PD-L1 drug, while pembrolizumab is an anti-PD-1 therapy. Nab-paclitaxel is a solvent-free, albumin-bound 130 nm particle form of paclitaxel (Abraxane, Abraxis Bioscience, Los Angeles, CA), which was developed to avoid toxicities associated with the Cremophor vehicle used in solvent-based paclitaxel, which is a nonneoplastic, cytotoxic, chemotherapeutic drug.

Results of Meta-Analysis

**Figure 5:**
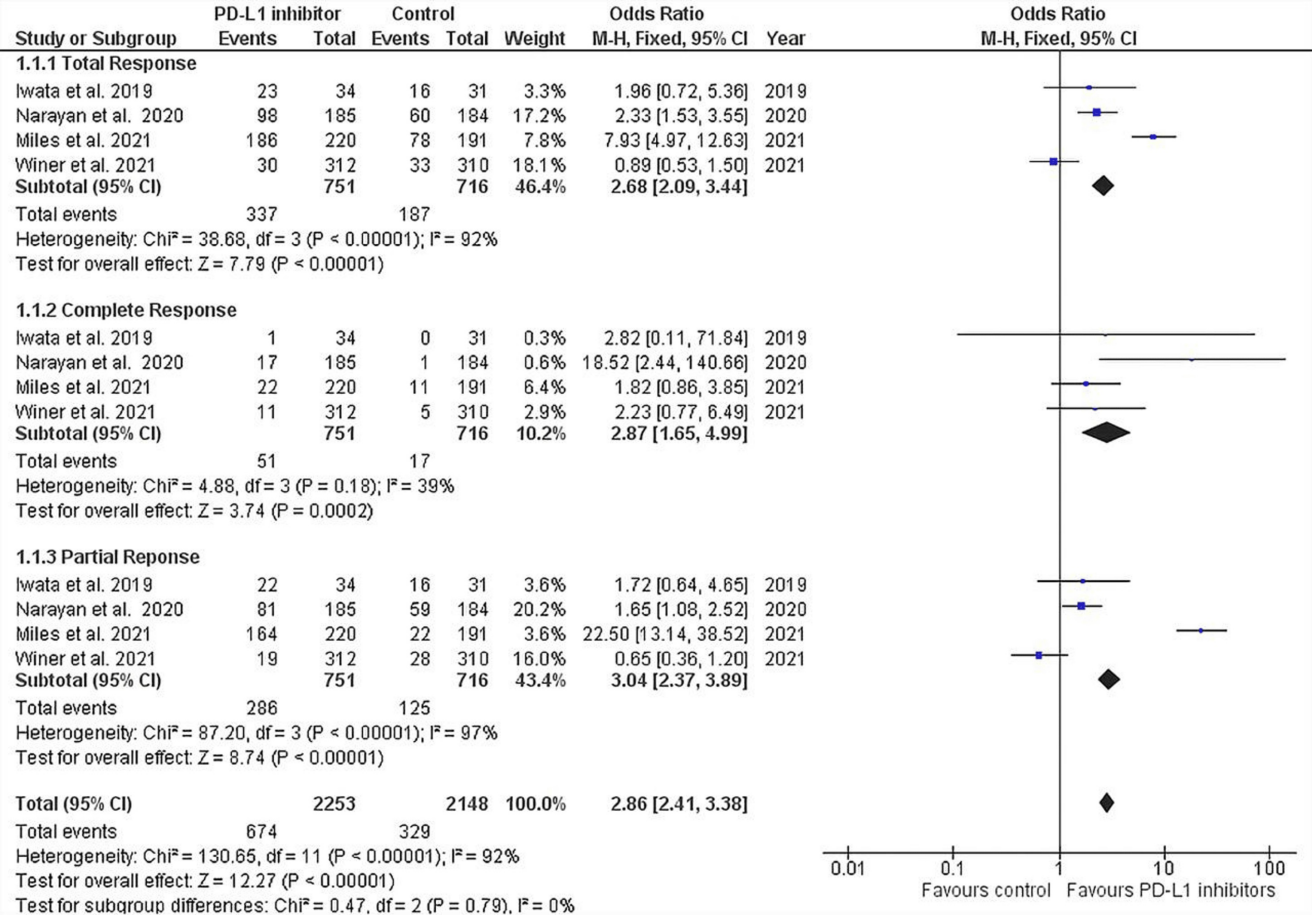
Forest plot for objective response Rate (ORR) for each included study This forest plot shows complete response, partial response, and total response of the treatment checkpoint inhibitor group versus control, which is the comparator group, favoring the checkpoint inhibitor group in all three responses [37-39,42] PD-L1, programmed cell death ligand 1; CI, confidence interval; df, degrees of freedom

Objective response rate: ORR was further divided into complete responses and partial responses and total responses (complete or partial). Four out of the six trials reported all these outcomes (Figure 5) [37-39,42].

Total response in the groups treated with anti-PD-1 or anti-PD-L1 therapies was calculated to be 44.87% PD-L1, as compared to the control group where the total response was 26.11%. The analysis revealed that the PD-1/PD-L1 inhibitor treatment group was associated with significantly higher total response in patients (OR, 2.68; 95% CI, 2.09-3.44; p < 0.00001) but with a high degree of heterogeneity between the studies (I^2^ = 92%). The heterogeneity dropped to 88% when we removed the study by Winer et al. (2021) [42] and to 75% when we removed the study by Miles et al. (2021) [38]. The heterogeneity dropped to 0% when both these studies were removed from the analysis. However, removing either or both of these studies also increased the p value to non-significance. The high heterogeneity suggests that there may be differences in participant characteristics (e.g., age, baseline disease severity, ethnicity, and comorbidities), types or timing of outcome measurements, and intervention characteristics (e.g., dose, frequency of dose, and training of interventionists). This shows that these studies are significant contributors to both the results and confounders.

Complete responses occurred in 51 out of 751 (6.79%) patients who received anti-PD-1 or anti-PD-L1 therapy, as compared to the control group where 17 out of 716 (2.37%) patients showed a complete response. Our meta-analysis revealed that the PD-L1 inhibitor group was associated with significantly higher complete response in patients (OR, 2.87; 95% CI, 1.65-4.99; p = 0.0002) with a low degree of heterogeneity between the studies (I^2^ = 39%). The low heterogeneity between the studies for this outcome measure, perhaps, reflects the increased objectivity associated with defining a complete response versus a partial response. The study by Iwata et al. (2019) has the largest confidence interval among all the studies, but removing it did not affect the significance of the results [39].

**Figure 6:**
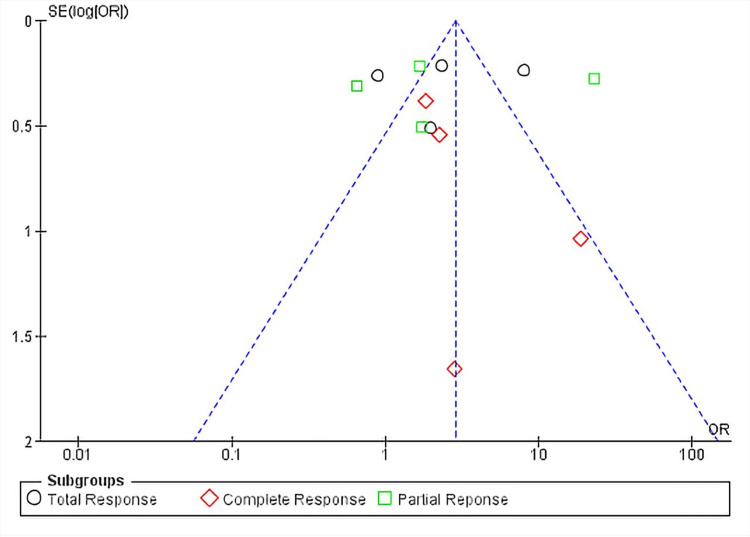
Funnel plot for ORR Funnel plot for ORR with the heterogeneity values of I^2^ = 92% for total response, I^2^ = 39% for complete response, and I^2^ = 97% for partial response in the checkpoint inhibitor group versus the control, which is the comparator group. Each plotted point represents the standard error (SE) and the standardized mean difference between the two groups. The triangle within the dotted plot lines represents the area where 95% of the data points would lie if there was an absence of publication bias. The vertical line within the triangle represents the average standardized mean difference ORR, objective response rate; OR, odds ratio

Partial response occurred in 286 out of 751 (38.08%) patients who underwent PD-L1 inhibitor, as compared to the control group where 125 out of 716 (17.48%) patients partially responded. The meta-analysis revealed that the PD-1/PD-L1 inhibitor group was associated with a significantly higher rate of partial response in patients (OR, 3.04; 95% CI, 2.41-3.38; p < 0.00001) but with a high degree of heterogeneity between the studies (I^2^ = 97%). Figure 6 shows the funnel plot depicting the heterogeneity between all studies. The heterogeneity remained 97% when we removed the study by Winer et al. (2021) [42] and dropped to 69% when we removed the study by Miles et al. (2021) [38]. The heterogeneity dropped to 0% when both these studies were removed from the analysis. However, removing either or both of these studies also increased the p value to non-significance. Removing the study by Miles et al. (2021) alone reduced the p value to 0.17, which indicates that it was the major contributor to the significance [38].

**Figure 7:**
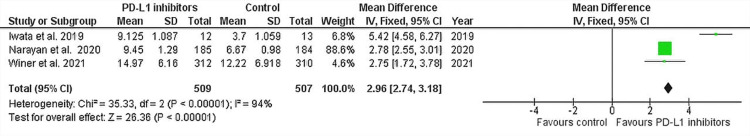
Forest plot for the duration of response Forest plot including the studies for the duration of response of the treatment checkpoint inhibitor group versus control, which is the comparator group, favoring the checkpoint inhibitor group in giving a favorable mean duration of response time [37,39,42] PD-L1, programmed cell death ligand 1; CI, confidence interval; df, degrees of freedom

**Figure 8:**
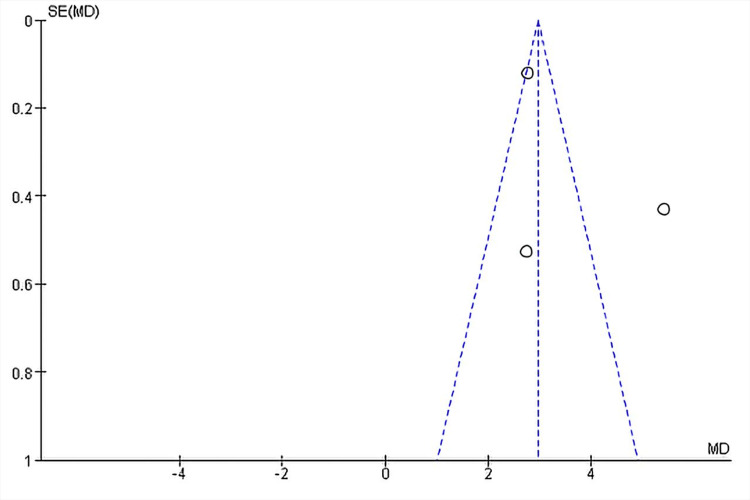
Funnel plot for the duration of response Funnel plot for the duration of response with the heterogeneity value of I^2^ = 94% in the checkpoint inhibitor group versus the control, which is the comparator group. Each plotted point represents the standard error (SE) and the standardized mean difference (MD) between the two groups. The triangle within the dotted plot lines represents the area where 95% of the data points would lie if there was an absence of publication bias. The vertical line within the triangle represents the average standardized mean difference

Duration of response: Three studies reported the DOR in months, which, on analysis, revealed a significant mean difference in the duration of response between the PD-1/PD-L1 group and the comparator arms [37,39,42]. Treatment with anti-PD-1 or anti-PD-L1 therapy significantly increased the mean duration of response (MD, 2.96; 95% CI, 2.74-3.18; p < 0.00001), showing less likely relapse in unit time on the PD-1/PD-L1 treatment versus the control containing treatment. However, this again had a high degree of heterogeneity between the studies (I^2^ = 94%) (Figure 7). Removing the study by Iwata et al. (2019) dropped the heterogeneity to 0% [39]. The high heterogeneity suggests that there may be differences in participant characteristics (e.g., age, baseline disease severity, ethnicity, and comorbidities), types or timing of outcome measurements, and intervention characteristics (e.g., dose, frequency of dose, and training of interventionists). The heterogeneity is shown in Figure 8.

**Figure 9:**
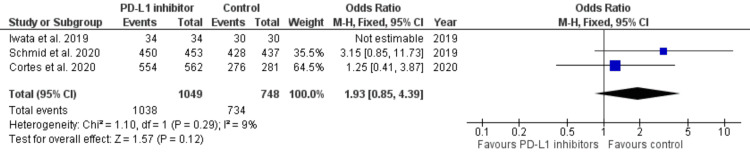
Forest plot for all-cause adverse effects Forest plot for all-cause adverse effects, including the studies in the treatment checkpoint inhibitor group versus control, which is the comparator group, showing no real significant difference in adverse effects in both groups [39-41] PD-L1, programmed cell death ligand 1; CI, confidence interval; df, degrees of freedom

All-cause adverse effects: Three out of six studies reported all-cause adverse effects [39-41]. All-cause adverse events were frequently observed in all arms of the studies; they occurred in 1,038 out of 1,049 (98.95%) patients in the PD-1/PD-L1 inhibitor group, while all-cause adverse events were observed in 734 out of 748 (98.12%) patients in the comparator arms. The analysis revealed that the PD-1/PD-L1 inhibitor group was not significantly associated with a higher number of all-cause adverse events (OR, 1.93; 95% CI, 0.85-3.87; p = 0.12) with no heterogeneity between the studies (I^2^ = 9%) (Figure 9). The frequency of adverse events was consistent in all the studies evaluated, and the findings reported here suggest that the addition of PD-1/PD-L1 inhibitors does not significantly increase the adverse event profile.

**Figure 10:**
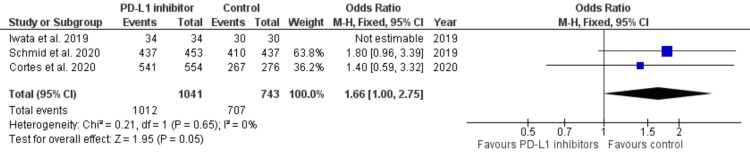
Forest plot for treatment-related adverse effects Forest plot for treatment-related adverse effects in the checkpoint inhibitor group versus the control, which is the comparator group, showing less treatment-related adverse effects in the control comparator group from the studies [39-41] PD-L1, programmed cell death ligand 1; CI, confidence interval; df, degrees of freedom

Treatment-related adverse effects: Three out of six studies reported treatment-related adverse effects [39-41]. Treatment-related adverse events occurred in 1,012 out of 1,041 (97.21%) patients in the PD-1/PD-L1 inhibitors group and in 707 out of 743 (95.14%) patients treated in the comparator arms. The analysis revealed that the PD-1/PD-L1 inhibitor group was associated with a significantly higher number of treatment-related adverse effects in patients (OR, 1.66; 95% CI, 1.00-2.75; p = 0.05) with no heterogeneity among the studies (I^2^ = 0%) (Figure 10). The reasons for the increase in treatment-related adverse events are likely due to the increased treatment burden in the PD-1/PD-L1 containing arms of the study; i.e., we might not expect the antibody control in the comparator arms to contribute to the adverse event profile.

Discussion

Immune checkpoint inhibitors function by eliminating the immune system’s negative control mechanisms that cancer cells exploit to evade recognition and destruction. By blocking the PD-1/PD-L1 interaction, these therapies restore immune system activity against tumor cells, and this form of immunotherapy has been approved for use in a number of cancer types, including breast cancer, as well as others [43,44]. Our study confirms that immune checkpoint inhibitors, atezolizumab or pembrolizumab, targeting PD-1 or PD-L1, have beneficial effects for patients with TNBC, as evidenced by increased response rates and the prolonged duration of response.

The approval of targeted treatments, such as the PARP inhibitors olaparib and talazoparib [45] and the PD-L1 inhibitor atezolizumab [37] for the treatment of advanced or metastatic TNBC, has stimulated the growth of studies to investigate their therapeutic potential in early-stage disease [46]. This underscores the importance of leveraging biomarker-driven strategies in treatment planning to maximize therapeutic efficacy. Despite this promise, the lack of standardized thresholds for PD-L1 expression complicates its use as a reliable predictor. ICIs such as pembrolizumab and atezolizumab have shown promising clinical efficacy in TNBC, with notable positive responses and survival rates for TNBC. Studies indicate median overall survival ranging from 11.2 to 18 months with pembrolizumab and 14.7 months with atezolizumab, along with ORR of up to 39.4% [47,48]. Combination therapy has also significantly improved progression-free survival (PFS) and OS [49]. These results emphasize the potential of checkpoint inhibitors for the treatment of TNBC by demonstrating positive clinical anticancer activity when administered alone or in combination. Results from our meta-analysis also show a similar trend.

Atezolizumab (MPDL3280A), a humanized IgG1 monoclonal antibody targeting PD-L1, demonstrated high specificity in binding to PD-L1 expressed on tumor cells [50,51]. Atezolizumab was evaluated in a phase I dose-escalation trial (NCT01375842) involving patients with solid malignancies with PD-L1 expression and was found to be effective in 100% and without treatment-related AEs in 5% of the population. The ORR was 19%, including two complete responses (CRs), two partial responses (PRs), and three cases of stable disease (SD). In patients with metastatic triple-negative breast cancer (mTNBC), atezolizumab was well-tolerated, and those with stable or responding disease and early treatment courses experienced sustained clinical benefits, consistent with our meta-analysis findings [18]. Contrary to expectations, high immune cell counts (>10%) were independently linked to better ORR and better OS [18].

Pembrolizumab (MK-3475), a monoclonal antibody targeting PD-1 on T cells, enhances immune responses by blocking the interaction between PD-1 on T cells and its ligand, PD-L1, found on tumor cells [48,52-54]. In the non-randomized phase Ib multi-cohort trial KEYNOTE-01231, 58.6% of 111 patients with metastatic TNBC had PD-L1-positive tumors. Patients received a median of five doses (range: 1-36 doses) of pembrolizumab. Grade 3 toxicity affected five patients (15.6%), with one treatment-related mortality. Common toxicities included arthralgia, fatigue, myalgia, and nausea. Median time to response was 17.9 weeks (range: 7.3-32.4 weeks), and the median duration of response was not yet reached (range: 15.0-47.3 weeks). The ORR among 27 evaluable patients was 18.5% [48].

First-line therapy with PD-1/PD-L1 inhibitors such as pembrolizumab and atezolizumab shows higher response rates and longer OS compared to their use in second-line or advanced treatment settings. Pembrolizumab exhibits an ORR of 21.4% and an OS of 18 months in first-line therapy versus an ORR of 5.3% and an OS of nine months in subsequent treatments [47,55]. Similarly, atezolizumab demonstrates an ORR of 24% and an OS of 17.6 months in first-line therapy, contrasting with an ORR of 6% and an OS of 7.3 months in later treatments [18]. These differences suggest potential evolution in TNBC and highlight challenges in maintaining immune response efficacy as treatment progresses, possibly due to tumor resistance mechanisms and chronic inflammation dynamics, which is referred to as “The Seventh Hallmark of Cancer,” overriding the positive effects of ICIs and decreasing their effectiveness [56].

PD-L1 expression on tumor cells is associated with better treatment outcomes and immune evasion through cytotoxic T cell inhibition. In contrast, PD-L2, with higher binding affinity to PD-1, has significant clinical implications, especially in TNBC, where elevated PD-L2 bearing extracellular vesicle (PD-L2EV) levels correlate with worse survival outcomes. Both biomarkers should be considered independently for precise patient stratification and therapy optimization [14,48,57,58]. Recent studies highlight the prognostic significance of PD-L1 expression in metastatic TNBC. A meta-analysis by Khan et al. demonstrated that PD-L1 expression strongly predicts objective response, one-year PFS, and two-year OS [59]. Similarly, Zhang et al. reported comparable pathological complete response (pCR) rates between PD-L1-positive and PD-L1-negative groups, with longer PFS observed in the combination treatment group [60]. Other studies, including a meta-analysis by Wang [61] and additional investigations, also highlight the close association between PD-L1 expression and the efficacy of PD-1/PD-L1 inhibitors in TNBC treatment [62,63], emphasizing the importance of patient stratification based on PD-L1 status.

Several studies that did not meet the inclusion criteria showed similar findings when comparing pembrolizumab plus chemotherapy to placebo or chemotherapy alone, with significant and clinically meaningful improvements [18,29,30,32,34,47,48,55,64-70]. However, some studies reported contrasting results. Miles et al. found no improvement in PFS or OS when paclitaxel was combined with atezolizumab [38]. Gianni et al. reported no improvement in pathological complete response (pCR) with the addition of atezolizumab to nab-paclitaxel, though PD-L1 expression was the key factor influencing pCR rates [63]. Wang (2022) in a meta-analysis showed that the use of PD-1/PD-L1 inhibitors had no significant effect on PFS or OS, but promising results were observed in the PD-L1-positive subgroup, where both PFS and OS improved significantly [61].

Adverse effects and treatment-related side effects have been reported in various trials, making the side-effect profile an important consideration [29,42,47,65,66,69-71]. Poor clinical prognostic factors, such as visceral disorders, liver metastases, multiple metastatic sites, and elevated lactate dehydrogenase levels, are associated with reduced response to checkpoint inhibitors and rapid disease progression [18,48,55]. To increase the therapeutic response rate in these contexts, alternative therapy approaches, as well as combination therapy, should be taken into account [55]. PD-1/PD-L1 inhibitors such as atezolizumab and pembrolizumab, added to comparator treatments, contribute to more adverse events, stemming from both typical side effects and those related to the additional medication. Most adverse events result from immune system activation by PD-1/PD-L1 inhibitors, with immune-related adverse events (irAEs) being of particular interest, consistent with previous studies on checkpoint inhibitors [72,73].

This study’s strengths lie in its use of phase III trials and robust statistical methods and focus on two FDA-approved agents. Limitations include a small sample size, potential publication bias, and variability in treatment durations and control arms, which may impact generalizability. High heterogeneity in efficacy metrics reflects the complexity of TNBC and the need for personalized treatment approaches. This review adheres to a predefined search and analytic framework; therefore, additional post hoc literature updates, sensitivity analyses, and statistical expansions were not undertaken. Despite these limitations, the findings suggest that PD-1/PD-L1-targeting treatments are promising for TNBC, warranting further large-scale randomized controlled trials.

Future research should standardize treatment protocols, identify biomarkers, and explore combination therapies to enhance efficacy and combat resistance. Genomic profiling and long-term studies are needed for deeper insights into mechanisms and survival. Investigating immune checkpoint inhibitors in early-stage TNBC, alongside therapies such as PARP inhibitors, may improve outcomes. Emerging technologies can reveal the tumor microenvironment’s role in resistance.

## Conclusions

Immune checkpoint inhibitors have a great deal of promise as a new treatment for TNBC, according to our systematic review and meta-analysis. Checkpoint inhibitors were shown to have effective anticancer properties in clinical studies, as evidenced by significantly increased response rates and duration of responses. Immune-related side effects of PD-1/PD-L1-blocking treatments are common but generally manageable, making them an acceptable risk. We anticipate that this finding will act as a springboard for more research on checkpoint inhibitor immunotherapy, revealing its promise as an efficient and acceptable treatment for breast cancer, particularly TNBC.
